# Effects of Supplementation with Goat Transitional Milk on Mortality, Growth, Rectal Temperature, and IgG Serological Level in Low-Birth-Weight Piglets

**DOI:** 10.3390/ani15121786

**Published:** 2025-06-17

**Authors:** Mónica Marcela Segura, Silvia Martínez-Miró, Miguel José López, Josefa Madrid, Verónica González, Fuensanta Hernández

**Affiliations:** 1 Department of Animal Production, Faculty of Veterinary, Campus Mare Nostrum, University of Murcia, 30100 Murcia, Spain; monicamarcela.segurar@um.es (M.M.S.); mjlopeza@um.es (M.J.L.); alimen@um.es (J.M.); nutri@um.es (F.H.); 2 Grupo de Investigación en Biodiversidad y Genética Molecular (BIOGEM), Faculty of Agricultural Sciences, Medellín Campus, Department of Animal Production, Universidad Nacional de Colombia, Carrera 65 #59A-110, Medellín 050034, Colombia; vgonzal@unal.edu.co

**Keywords:** caprine transitional milk, low-birth-weight piglets, nutritional supplementation

## Abstract

The increasing use of hyperprolific sows has led to greater litter heterogeneity, highlighting the need for effective nutritional support for low-birth-weight piglets. This study explores the potential of using surplus goat transitional milk as a nutritional supplement for piglets, especially for those facing increased survival and developmental challenges during the early life stages. To this end, two experiments were carried out as follows: in the first, goat transitional milk was administered via an oro-esophageal feeding tube to all piglets in the litter; in the second, concentrated transitional milk was administered via an oral dispenser to whole litters composed of low-birth-weight piglets. Performance parameters, mortality, and serum IgG levels were measured. In Experiment 1, supplementation with goat colostrum showed a tendency to improve weight in piglets ≤ 1100 g, with no significant effect on temperature, serum IgG level, or mortality. In Experiment 2, supplementation with goat colostrum did not affect live weight, rectal temperature, or serum IgG levels at 10 and 21 days. However, there was a trend towards lower mortality at 21 days in the supplemented piglets. These results suggest that goat transitional milk may provide context-dependent benefits, with a potentially greater impact on vulnerable piglets in more difficult conditions. Furthermore, its use represents a sustainable strategy to valorize surplus milk from goat farms.

## Simple Summary

This study evaluates the use of surplus goat transitional milk as a nutritional supplement to improve piglet survival and early development. Two experiments were conducted as follows: in one, all piglets in the litter were given goat transitional milk, while in the other, only low-birth-weight piglets were given goat transitional milk. The effects varied according to litter management, with a trend towards increased weight gain in lower-weight piglets when they were in high competition for maternal colostrum, while a trend towards decreased mortality was observed in supplemented piglets in litters consisting only of low-birth-weight piglets. These results demonstrate that goat transitional milk is a promising alternative for low-birth-weight piglets, especially in more difficult situations.

## 1. Introduction

Colostrum is a nutrient-rich fluid produced by mammals shortly after parturition, containing essential nutrients, immunoglobulins, and bioactive compounds which are critical for neonatal development. Bioactive peptides play a key role in protecting neonates from pathogens and stress while supporting metabolic regulation and improving physical and cognitive performance [1,2]. In piglets, adequate colostrum intake is vital not only for energy supply but also for passive immunity transfer, as the epitheliochorial nature of the porcine placenta prevents the transfer of IgG from mother to fetus [3]. This means that piglets rely entirely on colostrum to acquire immunity and energy during their first hours of life. However, the concentration of immunoglobulins in colostrum is highest in the hours before birth and decreases significantly within the first 24 h postpartum [4]. Likewise, the piglet’s gastrointestinal tract can only absorb intact macromolecules, such as immunoglobulins, for a short period after birth, highlighting the importance of rapid and adequate colostrum consumption to improve survival chances. [5]. In addition, newborn piglets are born with limited energy reserves, making colostrum essential for thermoregulation and physiological stability [6].

One of the main challenges of modern pig production is the management of piglets with low birth weights due to high prolificacy. Large litters often result in heterogeneous birth weights, increasing mortality around farrowing, and leading to complications such as delayed weaning, which negatively affects both productivity and animal welfare [7]. These problems are exacerbated when smaller piglets do not receive enough colostrum, compromising their survival and making them more vulnerable to hypothermia, disease, and infection. Therefore, ensuring that all piglets receive sufficient colostrum is essential for improving survival rates and optimizing performance in pig production systems.

Porcine immunoglobulin supplements have been used to improve growth and survival in colostrum-deprived piglets. However, it is unusual for sow farms to have a surplus of colostrum, as hyperprolific sows do not increase their colostrum production proportionally to the growth in litter size [8].

Other supplements have been studied in newborn piglets. Bovine colostrum has proven to be a promising option due to its high content of immunoglobulins, growth factors, and other bioactive components that support immune function and promote intestinal development. Studies have shown that bovine colostrum induces a systemic IgA response, enhancing resistance to infections, and has a positive effect on growth performance and feed efficiency [9,10].

Dairy goat milk products have rarely been considered for neonatal piglet supplementation; however, compared to cow’s milk, goat milk has smaller fat globules and higher protein digestibility, making it more easily absorbed and better suited for disease prevention. Additionally, goat milk is more alkaline and has greater buffering capacity [11]. Its medium-chain fatty acids offer antibacterial and antiviral benefits while providing a quick energy source for neonates [12].

Healthy goats often produce more colostrum than their offspring require, with around 3 L in the first 2 days [13]. Newborn kids consume only about 10% of their body weight in colostrum during their first feeding [14], approximately 246 mL for a Murciano–Granadina goat. In intensive dairy goats with artificial lactation systems, it is common to use only colostrum from the first milking for kids, and the rest of the colostrum is often discarded. The colostrum from the second milk is considered transitional milk (TM) and is not a marketable product on dairy farms; yet, it contains a high nutritional value, including immunoglobulins and bioactive compounds [15]. The use of goat TM in the supplementation of piglets represents a possible strategy to improve survival rates and performance in swine production and could have both economic and animal welfare benefits. However, its use in piglets remains largely unexplored.

Our hypothesis was that providing piglets with goat TM would deliver crucial energy and bioactive compounds, thereby supporting intestinal maturation and enhancing survival rates, especially in intrauterine growth-restricted piglets. This study aims to investigate the effects of supplementing neonatal piglets with goat TM collected during the second postpartum milking of Murciano–Granadina goats. More specifically, the use of goat TM as a nutritional supplement for neonates in the entire litter (Exp. 1) or for low-birth-weight piglets only (Exp. 2) is explored herein.

## 2. Materials and Methods

The study protocol was approved by the Ethics Committee for Animal Experimentation of the University of Murcia and the relevant administrative authority (Consejería de Agua, Agricultura, Ganadería, Pesca, Medio Ambiente y Emergencias, Region of Murcia, License NºA13220603).

### 2.1. Animal Housing and Management

This study was conducted on a commercial farm system (Huércal Overa, Almería, Spain) housing 4000 sows. All sows included in this study had the same genetic background (Large White × Landrace) and were raised under standardized housing conditions. The sows were transferred to the farrowing unit approximately at day 110 of gestation. A strict all-in-all-out management system was implemented, where the facility was thoroughly cleaned, disinfected, and left empty for up to six days before the introduction of new sows.

The farrowing unit featured free farrowing pens measuring 2.0 × 2.5 m with partially slated floor, maintained in a climate-controlled environment. The ambient temperature was set at 20 °C, with ventilation provided via negative pressure through wall inlets. In addition, each pen contained a heat source for the piglets. During gestation, the sows were fed on a barley and soybean meal-based diet (12.2 MJ/kg metabolizable energy (ME), 13 mg/kg crude protein, and 0.6 mg/kg lysine), offered at 2.5–3.0 kg per animal per day. After farrowing, the sows were transitioned to a commercial lactation diet (12.9 MJ ME/kg, 16 mg/kg crude protein, and 0.8 mg/kg lysine), beginning with 1.5 kg per feeding twice daily on the day after farrowing. Feed amounts gradually increased by approximately 0.5 kg per day based on the sows’ feed intake and recovery post-parturition. The diets were formulated according to the nutritional requirements of breeding sows (FEDNA) [16]. Sows had ad libitum access to water. Piglets were provided with a commercial creep feed starting on day 10 of life.

### 2.2. Collection and Processing of Goat Transitional Milk

The TM used in this trial was obtained from Murciano–Granadina goats housed on a livestock farm in Mula (Murcia, Spain). It was collected at the second postpartum milking (48 h) and had the following composition: 221 g dry matter/L, 74 g protein/L, 91 g fat/L, 38 g lactose/L, and 16 g IgG/L. Pasteurization was carried out by heating the milk in a water bath at 52 °C for 80 min. Following pasteurization, the TM was frozen at −20 °C. One portion was stored in its frozen state, while another was lyophilized for experimental use.

### 2.3. Experiments

Two experiments were conducted to evaluate the effect of goat TM supplementation on piglet performance in the farrowing unit. In Experiment 1, goat TM was administered via an oro-esophageal feeding tube system to all piglets in the litter, which were matched by average weight.

In Experiment 2, goat TM was concentrated and administered orally using oral dosing, but only to low-birth-weight piglets within the litter matched for low birth weight.

#### 2.3.1. Experiment 1

A total of 192 piglets from 16 multiparous sows of the same batch were used in this study. The average parity of the sows was 3.6 ± 0.37 (ranging from 1 to 6), and the mean body condition score was 3.5 ± 0.16.

Piglets from litters with a birth weight of 1700 g or less and without visible defects (such as splay legs or external anomalies) were included in this study. Within the first hour (time 0 h) after the completion of farrowing (placenta expulsion), each sow (according to parity and nutritional status) and its corresponding litter were randomly allocated to one of two treatment groups according to the oral supplementation of the piglets: the control group (1C) did not receive oral supplementation, and the goat group (1G) was orally supplemented with two doses of 20 mL of goat TM (approximately 4 g dry matter (DM) per intake) according to that used by Martinez-Miro et al. [17]. The goat TM was administered at 0 and 6 h after the end of farrowing. Goat TM was thawed under refrigeration and pre-warmed to 35 °C in a water bath before its administration to the piglets using a flexible oro-esophageal tube.

All piglets were individually identified with an ear tag clipped in the right ear. At 24 h post-farrowing, litters were standardized to 12 piglets through cross-fostering within each treatment group. Litter equalization was performed by ensuring that litters from each treatment had comparable mean birth weights and similar weight distributions. Regardless of supplementation, all animals were managed according to standard piggery routines.

Sow backfat and loin thickness were measured by ultrasound at entry into the farrowing room using a SF-1 Wireless Backfat & Loin Depth Scanner (Importvet, Barcelona, Spain), which is a B-mode ultrasound device equipped with a 45 mm, 5.0 MHz linear-array transducer. The images were obtained by placing the probe three times at 5 cm lateral to the dorsal midline, perpendicular to the loin behind the last rib; ultrasound gel was used as a sound-conducting material to achieve a better contact surface between the probe and the skin. At farrowing, the numbers of piglets born alive, stillborn, and mummified, as well as the body weights (BWs) of individual piglets born alive, were recorded.

In piglets, rectal temperature at 0 h and 6 h was recorded with a digital thermometer, prior to supplementation administration. The thermometer was inserted approximately 1 cm until a constant temperature was reached. Furthermore, mortality during the first 10 d of life was recorded. The piglets were weighed again on day 10 of life and the weight gain was calculated.

Blood samples (2 mL) were taken from the jugular vein of 20 piglets per treatment at 3 days of life into tubes without anticoagulant treatment and centrifuged for 15 min at 3000× *g*. The serum samples were stored at −80 °C until IgG analysis.

#### 2.3.2. Experiment 2

A total of 200 piglets weighing ≤ 1100 g BW from 80 multiparous sows from the same farrowing batch were used. The average piglet weight was 911.00 ± 148.17 g, with a range that varied from 490 g to 1100 g. The distribution of the population according to percentiles was as follows: 25% of the piglets weighed less than 810.0 g, the median value (50th percentile) was 950.0 g, and 75% of the piglets weighed less than 1037.5 g.

Within the litters, the selected piglets were randomly allocated to two treatments according to oral supplementation: a control group (2C) without oral supplementation and a goat group (2G), which received oral supplementation with concentrated goat TM in two intakes at 0 and 6 h after the end of farrowing. Prior to its administration, the freeze-dried goat TM was reconstituted in a 1:1 ratio with hot water (45 °C), thus reaching a five-fold concentration. Goat TM was fed to the piglets using an oral doser delivering 2 mL per pulse, with two pulses administered per intake. In this way, the animals were administered the same amount of goat TM (as DM) as in Exp. 1 (4 g DM per intake) but in a smaller volume per piglet (4 mL per intake). With this new methodology, it was possible to optimize the technique of goat TM feeding so that it could be incorporated into the farrowing management routine at any farm.

Each piglet was individually identified by means of an ear tag placed on the right ear. At 24 h post-farrowing, litters were standardized to 12 piglets through cross-fostering, taking into account the size of the piglets (a total of 17 sows were used for piglets weighing 1100 g or less in both treatments). In addition, care was taken to ensure that there was the same proportion of piglets from both treatments in all litters.

Piglets were weighed individually at 0 and 10 days of life, as well as at weaning (21 days). Rectal temperature (0 and 6 h) and mortality were controlled for 21 days. For IgG determination, blood samples (2 mL) were taken from the jugular vein of 20 piglets per treatment at 10 days of life into tubes without anticoagulant treatment. The samples were processed as described in Exp. 1.

### 2.4. Chemical Analysis

In piglet serum samples, goat and pig immunoglobulin (IgG) concentrations in Exp. 1 and pig IgG concentrations in Exp. 2 were measured using commercial pig- and goat-specific IgG indirect enzyme-linked immunosorbent assay (ELISA) kits (Bethyl Laboratories, Inc., Montgomery, TX, USA; references E101–104 and E50–104, respectively). In both kits, the detection range was 7.8–500 ng/mL The assays showed intra-assay coefficients of variation below 15%. The blinding method was used to analyze the results.

### 2.5. Statistical Analyses

All statistical analyses were performed using the IBM SPSS Statistics 28.0 system (IBM Corporation, Armonk, NY, USA).

In Exp. 1, data performance parameters (sows and litters) were analyzed using an independent samples *t*-test. Furthermore, piglets were classified into two groups according to their birth weight (≤1100 g and >1100 g), and a one-way analysis of variance (ANOVA), using litter as a random effect, was performed to study the effect of goat TM supplementation on the performance and temperature of piglets in each group. The experimental unit was litter, except when data were analyzed by categorizing piglets into two groups based on their birth weight, which is where each piglet represented an experimental unit.

In Exp. 2, each piglet was the experimental unit, and data on body weight and temperature were analyzed using a one-way analysis of variance (ANOVA) with the sow as the random effect. For both analyses, a Tukey test was performed to study possible differences between treatments.

In both experiments, the mortality rate was analyzed using a chi-square test and the serum IgG concentration data were analyzed using an independent samples *t*-test. In all statistical analyses, data are expressed as means, and differences were taken to be significant at *p* < 0.05, while those with 0.05 < *p* < 0.10 were considered near-significant trends.

## 3. Results

### 3.1. Experiment 1

#### 3.1.1. Sow Reproductive Performance

The descriptive characteristics of performance parameters for both gestating sows and their litter are presented in Table 1. No differences were observed between the control (1C) and supplemented group (1G) for any of the evaluated parameters (*p* ≥ 0.05), including backfat thickness, loin depth, total number of piglets born per litter, number of piglets born alive per litter, total weight of piglets born alive, and average weight of piglets born alive. These results indicate that the initial characteristics of sows and their litter were comparable between treatment groups.

#### 3.1.2. Piglet Performance

Table 2 summarizes the effects of goat TM supplementation on body weight and rectal temperature at 0 and 6 h after birth of piglets. The percentile analysis illustrates the distribution of piglet weights between the 1C and 1G treatments. At the 25th percentile, the piglets weighed less than 1122.5 g in group 1C and 1107.5 g in group 1G. The 50th percentile, representing the median, indicated maximum weights of 1290.0 g in group 1C and 1315.0 g in group 1G. At the 75th percentile, the piglets weighed up to 1477.5 g in group 1C and 1497.5 g in group 1G. These values reflect comparable weight distributions between the two groups of treatments.

Goat TM supplementation did not affect (*p* ≥ 0.05) body weight, weight gain, rectal temperature at 0 and 6 h after the birth of piglets, or litter size (10 d), compared to the control group (1C).

Table 3 presents the analysis of the results with piglets categorized into two groups based on their birth weight: ≤1100 g and >1100 g. There were no differences (*p* ≥ 0.05) between the 1C and 1G treatments in terms of birth weight, rectal temperature, and mortality rates in any of the birth weight categories. However, in piglets with birth weight ≤ 1100 g, supplementation tended to increase the final weight (*p* = 0.056) and weight gain (*p* = 0.065) at 10 days.

#### 3.1.3. Immunoglobulin G

Serum concentrations of IgG in piglets supplemented with goat milk on day 3 of life are presented in Table 4. Piglets in the 1G group showed (*p* < 0.05) higher serum goat IgG levels (0.7 ± 0.36 mg/mL) compared to the 1C group (0.2 ± 0.01 mg/mL). In contrast, no differences (*p* ≥ 0.05) were observed in serum pig IgG levels between the treatment groups.

### 3.2. Experiment 2

The effects of supplementation with concentrated goat TM on body weight, body temperature, IgG levels, and mortality in piglets with birth weight ≤ 1100 g are presented in Table 5. Supplementation had no impact (*p* ≥ 0.05) on body weight, weight gain, rectal temperature, or serum IgG levels at 10 or 21 days; mortality tended (*p* = 0.083) to be lower in group 2G (22%) compared to group 2C (34%).

## 4. Discussion

In pig farming systems using hyperprolific sows, the inverse relationship between prolificacy and birth weight represents a significant challenge. Larger litter sizes are often associated with a reduced average birth weight of piglets due to uterine constraints and competition for intrauterine nutrients [18,19]. This negatively impacts neonatal survival, as low-birth-weight piglets exhibit higher susceptibility to mortality, diminished vigor, and impaired postnatal growth [20]. Adequate colostrum intake is critical for the survival of these vulnerable piglets; however, the large litter sizes in hyperprolific systems frequently hinder the availability and proper distribution of colostrum, resulting in an insufficient intake of immunoglobulins and essential nutrients. Previous studies [21] highlight the importance of colostrum intake in pig production, showing its impact on production parameters and piglet mortality from birth to 22 weeks of age. Colostrum intake was the most relevant variable, correlating positively with weight at weaning, intermediate weight, and slaughter weight, especially in low-birth-weight piglets. To mitigate these issues, prior research has explored supplementation with porcine and bovine colostrum [22,23] or energy supplements [24,25,26] to compensate for inadequate colostrum intake.

In this study, Experiment 1, which included litters composed of both low- and normal-birth-weight piglets, showed that goat TM supplementation tended to enhance weight gain in piglets ≤ 1100 g. This suggests a potential beneficial effect of TM supplementation under conditions of intense competition for colostrum, in which low-birth-weight piglets are especially vulnerable due to reduced access to functional teats and stronger littermates. In such a scenario, early nutritional interventions like goat TM, rich in rapidly absorbable energy and bioactive compounds, may support vitality and growth. This result led us to consider Experiment 2 with a more representative sample size. In Experiment 2, where all piglets were uniformly of low birth weight (≤1100 g), these positive trends were not observed, even though TM was concentrated so that a smaller volume could deliver equivalent nutrients, aiming to minimize interference with natural suckling. This contrast in the results of both experiments may highlight the potential importance of litter composition and its management: when all piglets are of similar weight, competition for colostrum and teats is likely reduced, thereby diminishing the relative impact of supplementation. These findings support the notion that supplementing low-birth-weight piglets may be more effective in heterogeneous litters, where competition is more intense, as previously observed by Muns et al. [27].

Previous studies using colostrum supplements from different species have yielded mixed results regarding their impact on piglet growth. In this regard, Amdi et al. [28] showed that supplementation of intrauterine growth-restricted piglets with porcine colostrum was associated with a trend towards an increase in body weight at 8 h postpartum; however, they did not follow up the piglets afterwards. Martinez-Miró et al. [17] observed that supplementation with porcine or goat colostrum in newborn piglets did not affect piglet weight up to 20 days of age, but in this case, they did not use low-birth-weight piglets. In addition, bovine colostrum has been investigated as a possible substitute for or supplement to porcine colostrum. While some studies have reported improved immune responses and increased weight gain [10,23,29], others found no difference in the body weight or average daily weight gain of suckling piglets during the first 10 days of life under practical conditions, with a special emphasis on piglets with low birth weight [30]. The discrepancies observed among the referenced results may be attributed to variations in study conditions. Notably, some studies utilized bovine colostrum whey [29], while others employed powdered [10], whole colostrum [29] or bovine colostrum preparation [30]. Furthermore, differences in the age of the piglets used in each study could have influenced the outcomes, potentially accounting for the observed variability.

Neonatal thermoregulation is closely related to factors such as colostrum intake, genotype, environmental temperature, and vitality of the piglet at birth [31]. In the assessment of rectal temperature, this study found no significant differences between the supplemented and control groups in either experiment. Given that the ambient temperature was kept constant at 20 °C and heat sources were available to the piglets in both experiments, it is likely that TM supplementation alone was not sufficient to modify thermal parameters, especially in the absence of hypothermia. Many studies consider crushing to be the main cause of mortality, and it is common for weaker piglets to be placed close to the sow, which increases their risk of being crushed. However, crushing is often a consequence of hypothermia and starvation, with an interaction between these factors and crushing being a major contributor to neonatal mortality [25,32]. Supporting this interaction, Amdi et al. [28] reported that rectal temperature in neonatal piglets was influenced by both colostrum supplementation and the presence of the sow during the first hours of life. In their trial, piglets that received porcine colostrum showed higher rectal temperatures at birth, although this effect was transient and disappeared after 2 h. Similarly, piglets separated from the sow and subjected to external heat showed higher temperatures than piglets accompanied by sows at 2 h, but no differences were observed at 4 h. These results suggest that the thermoregulatory benefits of supplements or artificial heating may be short-lived unless combined with prolonged external contact or continuous thermal support.

Regarding mortality, the results of Experiment 2 showed a trend toward lower mortality compared to the control group. In high-risk populations, such as low-birth-weight piglets, even modest reductions in mortality can have considerable practical and economic relevance. These findings align with previous studies highlighting the potential of early oral supplementation. Muns et al. [25] observed a similar pattern toward reduced pre-weaning mortality in small piglets following the administration of oral bovine colostrum supplements within the first 12 h of life, along with improvements in immunological and endocrine parameters, without negatively affecting litter growth. In contrast to these results, we did not observe a clear effect of supplementation with goat TM on the piglets’ immunological parameters. As expected, goat IgG appeared in higher amounts in supplemented piglets; Martinez-Miró et al. [17] had already described that piglets absorbed goat IgG in the first hours postpartum, with an apparent coefficient of absorption similar to porcine IgG, although the possible effects of goat IgG on piglets are unknown. Regarding the level of pig IgG, it was similar in piglets from both treatments, both at 3 days postpartum in Exp. 1 and at 10 days postpartum in Exp. 2, despite the use of concentrated colostrum in Exp. 2 to reduce the volume administered, which did not affect endogenous pig IgG levels. These results show that supplementation with goat TM did not interfere with colostrum intake in piglets in both experiments. Furthermore, it should be noted that in piglets, the efficiency of IgG absorption after colostrum intake varies widely between individuals, even when doses are standardized. In addition, factors such as birth order, weight, and IgG concentration in maternal colostrum have a significant influence on resulting plasma levels, affecting neonatal immune competence [33].

Taken together, these results contribute to the ongoing discussion regarding colostrum supplements and their practical application in commercial swine systems. Goat TM—particularly that derived from surplus dairy production—emerges as a feasible, cost-effective, and potentially beneficial option, especially in situations where low-birth-weight piglets exhibit increased vulnerability due to limited access to colostrum or heightened competition within the litter.

The variability observed in the outcomes of neonatal supplementation highlights its high sensitivity to factors such as the timing and route of administration, volume supplied, and litter management [34,35]. Increasing evidence suggests that management decisions made within the first 24 h postpartum, including cross-fostering, may have a more decisive impact on piglet survival and growth than supplementation strategies alone [36]. Although cross-fostering is routinely employed to equalize litter sizes, its effectiveness largely depends on how it is applied and the conditions on each farm [27]. In addition, the implementation of “split suckling” or “split nursing”, the temporary removal of larger piglets or those that have already consumed colostrum, prior to fostering has been shown to enhance colostrum access for smaller or less vital piglets [37].

## 5. Conclusions

In conclusion, the use of goat TM as an oral supplement in low-birth-weight piglets showed context-dependent effects. In litters composed of both low- and normal-birth-weight piglets, TM supplementation tended to improve early weight gain in the smallest piglets (≤1100 g), suggesting a potential benefit under conditions of intense competition for colostrum. Conversely, in litters composed exclusively of low-birth-weight piglets, where competition was likely reduced, supplementation was associated with a trend toward decreased mortality. The variability observed across experiments underscores the complexity of neonatal piglet management. It also reinforces the idea that successful supplementation must be paired with optimal farrowing management practices, especially regarding litter equalization and early access to maternal colostrum. Therefore, the results of this study show that goat TM, particularly that derived from surplus dairy production, emerges as a feasible, cost-effective, and potentially beneficial option, especially in unfavorable contexts for piglets. However, it should be noted that this study has some limitations in that it does not evaluate long-term effects or effects under different contexts. Therefore, further studies are needed to optimize the use of TM as a supplement and to evaluate its effects in combination with other neonatal management practices in hyperprolific sow production systems.

## Figures and Tables

**Table 1:** Performance of gestation sows and litters of experimental groups of Exp. 1.

Item	Treatment ^1^		SED ^2^	*p*-Value
	1C	1G		
*n* (litters)	8	8		
Backfat thickness ^3^, mm	17.4	20.1	2.19	0.237
Loin depth ^3^, mm	52.7	50.6	2.94	0.468
Total number of piglets born/litter	14.9	15.9	1.39	0.483
Number of piglets born alive/litter	13.5	12.5	1.05	0.358
Total weight of born alive, kg	17.4	16.4	1.53	0.513
Average weight of born alive, kg	1.31	1.31	0.079	0.966

^1^ 1C, control group without supplementation; 1G, goat group supplemented with two doses (20 mL or 4 g of dry matter each) of goat transitional milk at 0 and 6 h after birth. ^2^ Standard error of difference (Student’s *t*-distribution). ^3^ Values of backfat and loin depth for sows at 110 days of gestation.

**Table 2:** Effect of goat TM supplementation on piglet body weight and rectal temperature across the different experimental treatments (Exp. 1) ^1^.

Item	Treatment		SED ^2^	*p*-Value
	1C	1G		
*n* (litters)	8	8		
Body weight (g)				
25th percentile	1122.5	1107.5		
50th percentile	1290.0	1315.0		
75th percentile	1477.5	1497.5		
Initial (0 h)	1270.9	1290.6	72.79	0.791
Final (10 d)	3334.3	3347.8	146.14	0.928
Weight gain (0–10 d)	2052.6	2043.7	101.69	0.932
Temperature after birth (°C) at				
0 h	37.6	37.8	0.21	0.415
6 h	37.9	37.8	0.20	0.663
Temperature increase (0–6 h)	0.27	0.05	0.215	0.329
Nº of piglets per litter (10 d)	11.4	10.9	0.51	0.173

^1^ 1C, control group without supplementation; 1G, goat group supplemented with two doses (20 mL or 4 g of dry matter each) of goat transitional milk at 0 and 6 h after birth. ^2^ Standard error of difference (Student’s *t*-distribution).

**Table 3:** Effect of goat TM supplementation on weight, rectal temperature, and mortality of piglets at 10 days, grouped by birth weight categories (≤1100 g and >1100 g; Exp. 1).

Items	Treatment ^1^		SEM ^2^	*p*-Value
	1C	1G		
Piglet birth weight ≤ 1100 g				
*n* (piglets)	22	24		
Body weight (g)				
Initial (0 h)	870.0	920.6	18.82	0.125
Final (10 d)	2541.0	2798.3	76.24	0.056
Weight gain (0–10 d)	1642.0	1876.4	72.54	0.065
Temperature after birth (°C)				
0 h	37.3	37.5	0.13	0.290
6 h	37.5	37.5	0.13	0.367
Temperature increase (0–6 h)	0.11	0.03	0.155	0.814
% Mortality (10 d) ^3^	9.09	8.69		1.000
Piglet birth weight > 1100 g				
*n* (piglets)	74	72		
Body weight (g)				
Initial (0 h)	1390.1	1413.9	14.54	0.552
Final (10 d)	3553.9	3511.7	42.69	0.856
Weight gain (0–10 d)	2161.4	2094.6	37.01	0.628
Temperature after birth (°C)				
0 h	37.7	37.8	0.06	0.461
6 h	38.0	37.8	0.05	0.131
Temperature increase (0–6 h)	0.27	0.05	0.059	0.114
Mortality (%, 10 d) ^4^	3.57	8.33		0.304

^1^ 1C, control group without supplementation; 1G, goat group supplemented with two doses (20 mL or 4 g of dry matter each) of goat transitional milk at 0 and 6 h after birth. ^2^ Standard error of the mean. ^3^ Chi-square (χ^2^) = 0.002; *p* = 1.000 (mortality of piglets ≤ 1100 g). ^4^ Chi-square (χ^2^) = 1.617; *p* = 0.304 (mortality of piglets > 1100 g).

**Table 4:** Concentration of goat and pig IgG in the serum of piglets at three days of age in the different experimental treatments (Exp. 1) ^1^.

Item	Treatment ^1^		SED ^2^	*p*-Value
	1C	1G		
*n* (piglets)	20	20		
Goat IgG (mg/mL)	0.2	0.7	0.21	0.039
Pig IgG (mg/mL)	52.7	59.2	5.41	0.240

^1^ 1C, control group without supplementation; 1G, goat group supplemented with two doses (20 mL or 4 g of dry matter each) of goat transitional milk at 0 and 6 h after birth. ^2^ Standard error of the difference (Student’s *t*-distribution).

**Table 5:** Effect of goat TM supplementation on weight, rectal temperature, and mortality of piglets with birth weight ≤ 1100 g at 10 and 21 days of age (Exp. 2).

Items	Treatment ^1^		SED ^2^	*p*-Value
	2C	2G		
*n* (piglets)	100	100		
Body weight (g)				
Initial (0 h)	896.0	926.0	11.90	0.253
10 d	2009.8	1973.4	46.53	0.916
21 d	3285.3	3157.3	85.24	0.751
Weight gain (g)				
0–10 d	1066.8	1028.5	41.57	0.848
0–21 d	2343.9	2213.9	82.28	0.919
Temperature (°C)				
0 h	36.9	36.9	0.11	0.927
6 h	37.5	37.5	0.07	0.327
Mortality (%, 21 d) ^3^	34.0	22.0		0.083
IgG (mg/mL, 10 d)	26.5	28.8	41.42	0.583

^1^ 2C, control group without supplementation; 2G, goat group supplemented with two doses (4 mL or 4 g of dry matter each) of goat transitional milk at 0 and 6 h after birth. ^2^ Standard error of difference (Student’s *t*-distribution). ^3^ Chi-square (χ^2^) = 3.001; *p* = 0.083.

## Data Availability

The data presented in this study are available upon request from the corresponding author (S.M.-M).
